# Streptavidin-Binding Peptide (SBP)-tagged SMC2 allows single-step affinity fluorescence, blotting or purification of the condensin complex

**DOI:** 10.1186/1471-2091-11-50

**Published:** 2010-12-31

**Authors:** Ji Hun Kim, Tsz M Chang, Alison N Graham, K HA Choo, Paul Kalitsis, Damien F Hudson

**Affiliations:** 1 Murdoch Childrens Research Institute, Royal Children's Hospital, Melbourne, Victoria 3052, Australia; 2 Department of Paediatrics, University of Melbourne, Royal Children's Hospital, Melbourne, Victoria 3052, Australia

## Abstract

**Background:**

Cell biologists face the need to rapidly analyse their proteins of interest in order to gain insight into their function. Often protein purification, cellular localisation and Western blot analyses can be multi-step processes, where protein is lost, activity is destroyed or effective antibodies have not yet been generated.

**Aim:**

To develop a method that simplifies the critical protein analytical steps of the laboratory researcher, leading to easy, efficient and rapid protein purification, cellular localisation and quantification.

**Results:**

We have tagged the SMC2 subunit of the condensin complex with the Streptavidin-Binding Peptide (SBP), optimising and demonstrating the efficacious use of this tag for performing these protein analytical steps. Based on silver staining, and Western analysis, SBP delivered an outstanding specificity and purity of the condensin complex. We also developed a rapid and highly specific procedure to localise SBP-tagged proteins in cells in a single step procedure thus bypassing the need for using antibodies. Furthermore we have shown that the SBP tag can be used for isolating tagged proteins from chemically cross-linked cell populations for capturing DNA-protein interactions.

**Conclusions:**

The small 38-amino acid synthetic SBP offers the potential to successfully perform all four critical analytical procedures as a single step and should have a general utility for the study of many proteins and protein complexes.

## Background

We have utilised the SBP tag to perform four separate cell biological and biochemical techniques on the SMC2 subunit of condensin, comparing the effectiveness of this technique using antibodies generated against SMC2. The SBP tag was originally isolated using mRNA display from an 88-amino peptide library designed to identify small peptides that bind streptavidin with high affinity [1,2]. Through N- and C- terminal deletion mutants the original streptavidin binding peptide, SB19, was truncated to a minimal 38 amino acid SBP tag (MDEKTTGWRGGHVVEGLAGELEQLRARLEHHPQGQREP), described here. The SBP has a dissociation constant K_d _of 2.5 nM to streptavidin and is eluted natively using biotin, allowing non-denaturing conditions for protein purification. The small size of this synthetic tag makes it amenable to tagging proteins *in vivo *without disrupting their function.

We have previously shown that SBP-tagged SMC2 protein of the condensin complex fully rescues function when stably expressed in conditional SMC2 knockout chicken DT40 cells [3]. In vertebrates, there are two condensin complexes which share the same core SMC subunits (SMC2 and SMC4), but differ in their three auxiliary subunits (CAP-D2, H, G for condensin I and CAP-H2, D2, D3 for condensin II). Both condensin I and II are essential for proper chromosome organisation and packaging during mitosis and meiosis [4,5].

The ability of condensin to behave as an enzyme capable of altering DNA topology [6] and the distinct cell cycle localisation of the complex to chromosomes [7,8] requires researchers to develop a system where the complex can be both purified natively and examined in cells using fluorescence imaging. We demonstrate here that the condensin complex can be purified to near homogeneity using the SBP tag in a single step. Furthermore this purity was not compromised when the cells were chemically cross-linked thus making it ideal for analysing attached DNAs. We have demonstrated for the first time that the SBP tag can be used to localise proteins in cells with fluorescence using a single-step streptavidin fluorescent conjugate. Detection of SBP-tagged SMC2 (SMC2-SBP) on mitotic chromosomes with a streptavidin-488 fluorophore displays the characteristic axial staining of chromosome scaffold proteins like the condensin complex. We have also shown the utility of the single step SBP affinity-fluorescence by applying it to SBP tagged CENP-A protein, which showed characteristic centromere staining and co-localisation with a CENP-A antibody. The ability to quantify from cell cultures and purify SBP-tagged proteins natively with biotin in a single step, in addition to the ease of detection on fixed cells makes the SBP tag one of the most effective affinity tags available.

## Methods

### Cell lines

DT40 wild-type clone 18 cells were isolated as described previously [9]. We have previously shown that SMC2-SBP expressed from a transgene containing the SMC2 endogenous promoter fragment can fully sustain viability of a Tetracycline (Tet)-repressible SMC2 knockout (KO) in DT40 cells [3,10]. In this cell line (simplified to SMC2-SBP cells for this study), the Tet-repressible SMC2 gene is switched off leaving the SMC2-SBP as the only form of SMC2.

### Culture conditions

DT40 cells were cultured in RPMI 1640 (GIBCO) supplemented with 10% FBS, 1% chicken sera and L-Glutamine. Cells were grown at 39°C in 5% CO_2_. SMC2-SBP cells were grown in 200 ng/ml doxycycline to ensure the tagged version of SMC2 was the only form being expressed.

### Detection of SBP on mitotic chromosomes

Wild type and SMC2-SBP cell lines were incubated with 75 mM KCl for 8 minutes at 37°C and cytospun onto slides at 700 rpm (medium acceleration) for 5 minutes. The slides were then flooded with KCM (120 mM KCl, 20 mM NaCl, 10 mM Tris/HCl pH 7.4, 5 mM EDTA, 0.1% Triton X-100) as described [11] and left at room temperature for 10 minutes. Alexa 488 streptavidin (Invitrogen) was diluted at 1/1000 from stock (2 mg/ml) in KCM and incubated at room temperature for 45 minutes. Slides were washed 3 × 5 minutes in KCM then incubated for 10 minutes in 4% paraformaldehyde in KCM, followed by three rinses with distilled water (dH_2_O). Vectashield containing DAPI (Vector Laboratories) was used to mount the slides and images were processed using a Zeiss Axioplan wide-field fluorescent microscope.

### Isolation of the condensin complex with SBP-tagged SMC2

SBP-tagged condensin was isolated from DT40 cells using streptavidin-sepharose beads (Pierce) with the following purification procedure. In general, 4 × 10^8 ^SMC2-SBP stably transfected DT40 cells were snap frozen as cell pellets and stored at -80°C. Lysis was performed on thawed cell pellets and resuspended in 50 mM Tris/HCl pH 7.4, 250 mM NaCl, 0.5% NP-40, 1 mM CaCl_2_, 30 μg/ml RNase A, and freshly added protease inhibitors (Roche) for 45 minutes on ice followed by the addition of 1 mM EDTA and 0.1% deoxycholate. Lysates were centrifuged at 4°C for 10 minutes at 14,000 rpm. Streptavidin-sepharose beads (600 μl) were mixed with cleared lysate (supernatant) for two hours whilst rotating at 4°C in a final volume of 10 ml. Beads were washed with wash buffer (50 mM Tris/HCl pH 7.4, 250 mM, 0.5% NP-40, 0.1% deoxycholate) three times and eluted in elution buffer (50 mM Tris/HCl pH 7.4, 250 mM NaCl 0.5% NP-40, 0.1% deoxycholate, 4 mM biotin) at 4°C for 1 hour.

### Silver staining analysis of purified proteins

Crude lysates and pull-down eluents from the above purification were boiled in SDS-sample buffer (Invitrogen) for 5 minutes at 95°C and stored at -20°C. The pull-down eluents were subjected to SDS-PAGE on a 4-12% BisTris gel (Invitrogen), followed by silver staining as described [12].

### Isolation of the cross-linked condensin complex with SBP-tagged SMC2

3 × 10^8 ^SMC2-SBP cells were cross-linked in 1% formaldehyde (Merck) for 10 minutes at room temperature followed by quenching in 125 mM glycine for 5 minutes in culture. The cell lysate was sonicated using the Bioruptor (Diagenode, Belgium) to shear the DNA between 100 - 500 bp before centrifugation. The cross-linked complexes of DNA and SMC2-SBP were purified and eluted from streptavidin beads as described above and the attached DNA extracted using phenol/chloroform followed by precipitation with isopropanol and resuspension in TE buffer (10 mM Tris-Cl, pH 7.5. 1 mM EDTA).

### Western analysis of SMC2-SBP

Equal protein amounts of crude lysate (input) and eluent were run on 4-12% BisTris gels (Invitrogen), followed by immunoblotting or affinity blotting. For immunoblotting, blots were probed with rabbit anti-SMC2 (M) antibody [13] for 2 hours at 1:2000 in 1% bovine serum albumin (BSA) PBS/0.2% Tween 20 (PBT) followed by anti-rabbit-HRP (Millipore) or alkaline phosphatase-conjugated antibody (Jackson Immunoresearch Laboratory) for 1 hour at 1:10,000 or 1:2000 in 1% BSA/PBT, respectively. For affinity blotting, blots were probed with HRP (Pierce) or AP (Millipore) conjugated streptavidin for 1 hour at 1:10,000 or 1:2000 in 1% BSA/PBT, respectively. HRP blots were analysed using a chemiluminescence kit (GE Healthcare, USA), whilst AP blots were developed by incubation in NBT/BCIP staining solution (6.1 μM NBT, 4.3 μM BCIP, 0.1 M Tris/HCl pH 9.5, 0.1 M NaCl, 5 mM MgCl_2_/6H_2_O) for 30 minutes followed by fixation in a stop solution (20 mM Tris/HCl pH 8, 5 mM EDTA).

### Quantification of SMC2 Recovery

Known protein amounts were subjected to SDS-PAGE in 4-12% BisTris gels (Invitrogen) and blotted as described above. The intensities corresponding to each antigen were quantified using ImageJ (NIH Image; http://rsb.info.nih.gov/ij/), expressed as relative SMC2 yield. The purification factor of SMC2-SBP in the single step purification was calculated as follows:

$$\frac{relative\;SMC2\;yield\;in\;eluent}{relative\;SMC2\;yield\;in\;input}\times \frac{amount\;of\;total\;protein\;in\;input\;lane}{amount\;of\;total\;protein\;in\;eluent\;lane}$$

## Results/Discussion

### SBP tag can be detected with a streptavidin fluorophore conjugate

**Figure 1:**
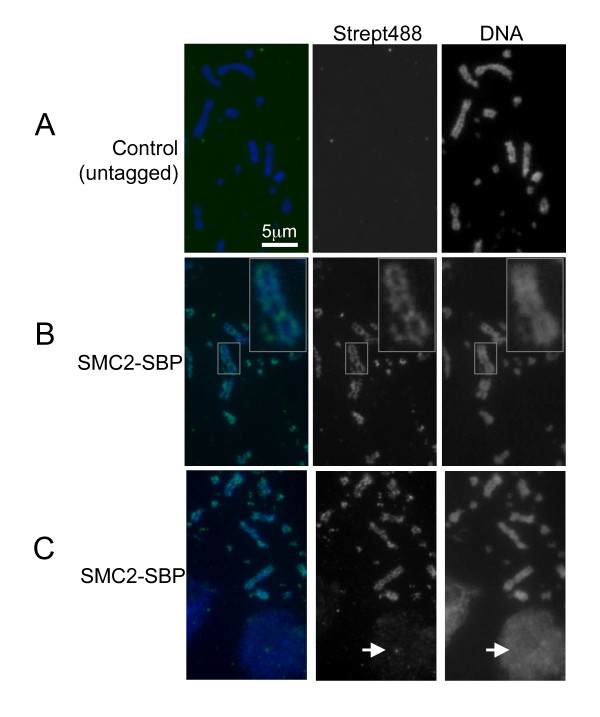
**Single step fluorescent detection of SBP-tagged SMC2**. Cyto-centrifuged DT40 wild-type cells (A) and SMC2-SBP cells (B, C) were incubated with streptavidin-488 for single-step fluorescence analysis. Mitotic chromosomes from DT40 wild-type cells show no staining, whilst chromosomes from SMC2-SBP display the distinct characteristic chromosome scaffold staining pattern of condensin subunits. Note also in (C) that the interphase nuclei display weak diffuse staining of SMC2-SBP cells which is also consistent with known condensin antibody staining [8].

We investigated whether we could detect SBP-tagged proteins in a single step using the streptavidin-Alexa 488 fluorophore. DT40 SMC2-SBP and wild-type cells were cytospun and incubated with streptavidin-488 as described in Material and Methods. The results indicated that a single-step labelling using streptavidin-488 gave the distinct axial staining characteristic of all the condensin complex members and other scaffold proteins (Figure 1B, C). Importantly, there was no chromosomal staining on the untagged DT40 cells, suggesting the staining was specific to SBP (Figure 1A). We found the staining using streptavidin-488 mimicked that obtained using anti-SMC2 antibody on DT40 wild-type cells (Additional file 1A) and in general the streptavidin-488 fluorophore showed less background and could be used at a greater dilution. We routinely used the streptavidin-488 fluorophore at 1/1000 (2 μg/ml) but noted strong signals at 1/2000 (1 μg/ml). There was little background and the interphase nuclei also stained albeit weakly which is consistent with results from both live imaging of GFP-tagged condensin and also using condensin antibodies on fixed cells [7,14]. We therefore conclude this technique is reliable and sensitive and believe it to be widely applicable for cellular localisation of SBP tagged proteins.

To show that the SBP tag was useful for the fluorescent detection of other proteins in other cell lines, we also created an SBP fusion to the centromere-specific protein, CENP-A. Expression of an SBP-Cenpa construct in mouse cells showed that the tagged protein was readily detected and specific to the centromere regions of the interphase nucleus (Additional file 2).

### Single-step purification of SMC2-SBP and associated condensin complex subunits

**Figure 2:**
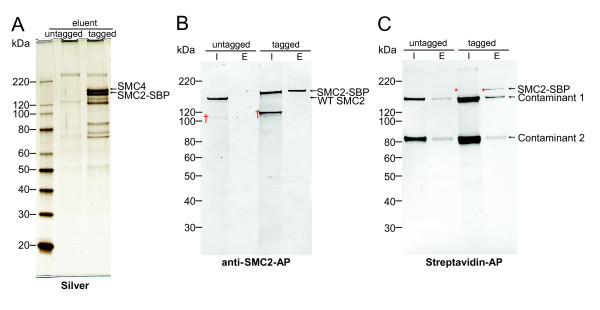
**Analysis of single-step purification of SBP-SMC2**. (A) Equivalent amounts of protein eluent from untagged DT40 wild type and SMC2-SBP preparations were analysed by silver staining. SMC2-SBP and its condensin dimerisation partner SMC4 appear as the predominant bands in tagged eluent. (B) Standardised protein amount of crude input (I) and eluent (E) of both untagged and SMC2-SBP were immunoblotted using rabbit anti-SMC2 antibody followed by anti-rabbit-AP to detect SMC2 proteins. SMC2-SBP is present in both E and I, whilst wild-type (WT) SMC2 is not present after purification. (C) Single step affinity blotting of SMC2-SBP. The same samples in (B) were affinity blotted using streptavidin-AP. SMC2-SBP is detected in both input, and eluent of the SMC2-SBP tagged extracts, whilst, not in untagged extracts. Streptavidin contaminants 1 and 2 are present in both untagged and tagged. Note ^† ^indicates SMC2 degradation product, and * indicates SMC2-SBP detected by streptavidin-AP.

Crude lysates of SMC2-SBP and also that of untagged wild-type DT40 were purified using streptavidin-sepharose to analyse the purification of SMC2-SBP and its associated proteins. Proteins in the eluent were detected by silver staining as described in Materials and Methods. The silver staining showed both SMC2-SBP and its dimerisation partner SMC4 present in near equimolar amounts, as would be expected from a functional condensin complex (Figure 2A). In contrast, the untagged wild-type lysate had barely detectable silver stain bands and none, which corresponded to condensin subunits (Figure 2A). This suggests that the single-step purification using streptavidin selectively enriches SBP tagged proteins and associates, but not the non-specific proteins.

### Western analysis of SMC2-SBP pull-downs

Purification and enrichment of SMC2-SBP from crude lysates was determined using colorimetric and chemiluminescence assays as described in the Materials and Methods. SMC2 was present in the input of both wild-type and SMC2-SBP cells. However, SMC2 was detected only in the eluent from SMC2-SBP cell extracts after the single-step purification (Figure 2B, Additional file 3A). Increasing the loading amount of the eluent on the gel 10-fold did not result in the detection of untagged SMC2 (data not shown). A degradation product (95 kDa) of SMC2 was detected in the input of both untagged and tagged cell extracts, though to a much lesser extent in the untagged cells as detected with anti-SMC2 antibody (Figure 2B, Additional file 3A). Earlier studies on chicken SMC2 have shown that the protein is very sensitive to proteolysis and frequently produces a ladder when blotted with anti-SMC2 antibody [13]. A possible explanation for the increased degradation in the transgenic line is the endogenous SMC2 locus may contain further regulatory elements necessary for precise timing and therefore stability for the SMC2 protein. Nonetheless, this product was washed out during the purification and thus absent in the eluent. Streptavidin-conjugate for single-step analysis also detected SMC2-SBP in both input and eluent, using either the Streptavidin-AP or Streptavidin-HRP conjugates (Figure 2C and Additional file 3B, respectively). For both conjugates, the SMC2-SBP was faintly detected in the input lanes but significantly increased in the elution lanes. Two contaminants appeared at 80 and 145 kDa in both DT40 wild type and SMC2-SBP cell extracts, which we believe to be binding to streptavidin non-specifically. Proteins of approximately the corresponding size of the contaminants also appear in the silver stain of the untagged at above 80 and 120 kd respectively (Figure 2A). Both contaminants decreased significantly following purification compared to the marked increase in SMC2-SBP (Figure 2C), implying significantly lower affinity of the contaminants for streptavidin. The high affinity of streptavidin to bind the SBP further supports affinity fluorescence data obtained using streptavidin-488, where no signal was detected on untagged cells (Figure 1A).

### Calculation of purification factor of SBP purified condensin

The purification factor of SMC2-SBP based on the anti-SMC2 blots (Figure 2B, Additional file 3A) was calculated as described in Materials and Methods. At equivalent SMC2-SBP signal in both input and eluent on Western blot (Figure 2B), the loading amount of protein for input and eluent were 26 and 0.013 μg, respectively, giving approximately 2000-fold enrichment in SBP-tagged protein after purification. Therefore we propose that the SMC2-SBP can be purified close to near homogeneity using the SBP tag. The signals of the two contaminants detected by streptavidin-conjugate blot decreased by 125-fold whilst protein loading amount decreased from 17 to 0.004 μg after single purification, giving increase in the enrichment of both around 30-fold (Figure 2C). This suggests highly selective purification of SBP-tagged proteins.

### Single step purification of cross-linked condensin complex

**Figure 3:**
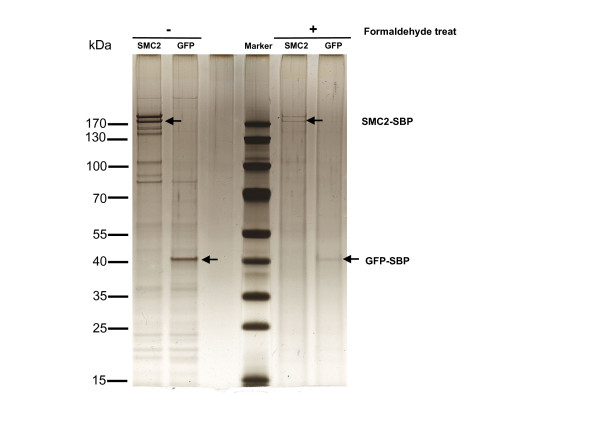
**Isolation of SBP-SMC2 from formaldehyde cross-linked cell populations**. Silver stain analysis of SBP tagged SMC2 and GFP isolated from uncross-linked (left panels) and cross-linked cell populations (right panel). SMC2 SBP isolated from cells cross-linked with formaldehyde show integrity of the SMC2-SMC4 heterodimer with no significant increase in background bands. Note the GFP-SBP also appears purified to near homogeneity from cross-linked cells but bound no significant amount of DNA. Only sample isolated from the SMC2-SBP purified cross-linked complexes contained significant amount of DNA, suggesting the SBP tag will be highly amenable for enriching DNAs of chromosomal proteins.

Next we looked at the effect of formaldehyde cross-linking on the SBP tag. For ChIP and other DNA analyses, chemically cross-linking a population of cells is often essential to ensure capture of complex associated DNA. A major limitation is that the cross-linking procedure inherently introduces more non-specific background and potentially interferes with affinity of the tag so we were interested to see how the SBP tag performed under these conditions. To analyse the SBP-associated DNAs, SMC2-SBP cells and GFP-SBP cells as a negative control (not expected to bind DNA), were cross-linked in culture using formaldehyde and purified over streptavidin-sepharose. Remarkably even after the formaldehyde treatment, both SMC2-SBP and SMC4 are present predominantly in the eluent with little increase in background compared to purified sample from uncross-linked (Figure 3). Using the same number of cells in the starting material the SMC2-SBP purified 2.38 ng DNA whereas the GFP SBP produced barely detectable DNA. This is consistent with condensin's known ability to bind DNA and also suggests the resulting DNA is likely to be highly specific. Therefore we believe the SBP tag is also ideal for isolating complex-associated DNAs.

## Conclusions

### Four-way analysis using SBP

The range of affinity and epitope tags available is ever increasing but few can be used efficiently for multiple techniques. For example, GFP technology is very convenient for fluorescent imaging but has no characteristic that allows straightforward purification aside from immuno-precipitation using anti-GFP antibodies which are considerably less efficient at isolating the tagged proteins and do not, like SBP, allow native purification of the complex. An important consideration is whether the tag can be fused to proteins without inhibiting function. For the condensin complex in DT40 cells, the SBP-tagged SMC2, fully complements SMC2 conditional knockout cells and is thus the only form of the essential protein present in the cells. The small size (38 amino acids, 4 kDa) of the SBP tag and the overall neutral charge [15] suggests it might be useful for complementing a wide range of genes in systems where the endogenous gene has been deleted or switched off.

A key advantage of SBP is that it can be eluted natively using biotin. This is particularly relevant to enzymes such as condensin or for complexes whose assays require native conditions for activity. Therefore the SBP tag is of great utility to both proteomics analysis and *in vitro *biochemical assays.

Other more commonly used tags such as S-tag, histidine and haemagglutinin require harsher or denaturing conditions for elution from beads (for review see [15,16]). This is disadvantageous for downstream biochemical analyses. A shorter eight amino acid Streptavidin affinity tag known as the Strep-tag I, and the improved higher affinity Strep II tag are also available [17,18]. These tags have the advantage of being small and are also eluted natively with biotin derivatives, but bind streptavidin less tightly with dissociation constants of 10-37 μM and 18-72 μM for Strep I and II, respectively, compared to SBP which has a dissociation constant of 2.5 nM. Furthermore, SBP is an improvement on other Biotin-Streptavidin affinity systems such as the biotin ligase purification system which requires additional proteins to be expressed concurrently (i.e., biotin ligase) in addition to the tagged target protein [19].

The ability to use the SBP tag to natively isolate highly purified proteins or complexes also allows detailed structural analysis as demonstrated elegantly when SBP is attached to clathrin light chain A (CLCA) and also type 1 ryanodine receptor (RyR1) [20]. Electron microscopy on the eluted structures shows clearly the preserved characteristic three armed pinwheel structure of CLCA and quatrefoil characteristics of RyR1 respectively. We also anticipate similar utility for SBP tagged condensin complexes.

The SBP tag has many advantages that makes it appealing to cell biologists: 1) the small tag size means it is unlikely to disrupt function, 2) it is able to purify complexes to near homogeneity under native conditions which are likely therefore to retain structural integrity, 3) SBP-tagged proteins can be directly visualised in fixed cells in a single step, 4) SBP tagged proteins can be readily enriched from cross-linked cell populations. This final application has important implications for the rapidly evolving next generation sequencing technology as applied to chromosomal proteins and transcription factors, allowing easier identification of protein bound DNAs to create genome-wide maps or define target motifs [21]. These qualities no doubt make it one of the most appealing and versatile affinity tags available to researchers.

## Authors' contributions

JHK designed and carried out experimental procedures, and drafted the manuscript; TMC and AG performed and analysed experiments; KHAC analysed data and contributed to writing; PK and DH contributed to experimental design, experiments and manuscript writing. All authors have read and approved the final manuscript.

## Supplementary Material

Additional file 1 **SMC2 and SMC2-SBP chromosome staining**. (A) Top panel shows DT40 wild-type metaphase chromosomes stained with an anti-SMC2 antibody showing enrichment of the axial region of the sister chromatids. (B) Streptavidin 488 similarly binds to the axial regions of the metaphase chromosomes. Click here for file

Additional file 2 **Cenpa and SBP-Cenpa interphase nuclear staining**. Mouse cells transiently transfected with an SBP-Cenpa construct exhibit punctate nuclear staining detected by streptavidin-488. These signals co-localise with a rabbit antibody that is specific for the mouse Cenpa protein. Click here for file

Additional file 3 **Quantitation of SMC2-SBP purification**. (A) Standardised amounts of input (I) and eluent (E) from both DT40 (untagged) and SMC2-SBP (tagged) protein extracts were immunoblotted with a rabbit anti-SMC2 antibody, followed by anti-rabbit-HRP. Similar to Figure 2B, SMC2-SBP is present in both I and E from tagged, whilst wild-type (WT) SMC2 is not present in E from untagged. Note ^† ^indicates SMC2 degradation. (B) Equivalent cell amounts (5 × 10^5 ^cells) of input and eluent from both untagged and tagged (I and E1, respectively) as well as 5 and 10-fold diluted eluents (E5 and E10 respectively) were affinity blotted with streptavidin-HRP. SMC2-SBP (*) is present in all input and eluents from tagged using streptavidin HRP. Streptavidin contaminants 1 and 2 were detected in all lanes. Click here for file
