# Supplementary figures and images for: Streptavidin-Binding Peptide (SBP)-tagged SMC2 allows single-step affinity fluorescence, blotting or purification of the condensin complex
Source: BMC Biochem. 2010 Dec 31;11:50. doi: 10.1186/1471-2091-11-50 (PMC3022668; doi:10.1186/1471-2091-11-50)

SMC2

DNA

A

control

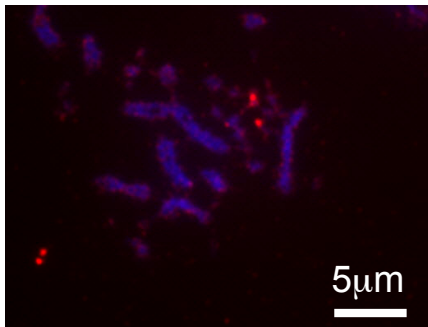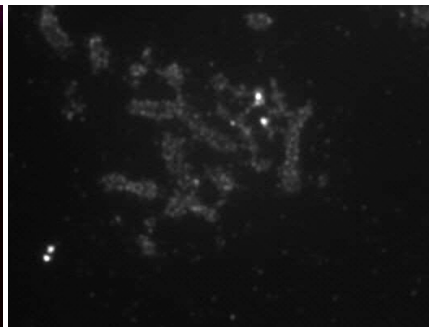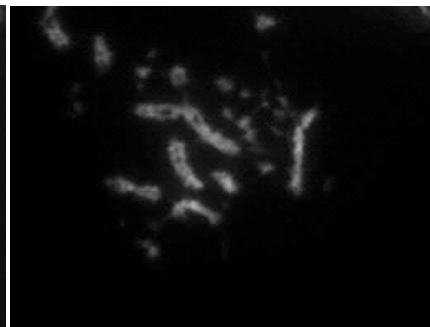

B

SMC2-SBP

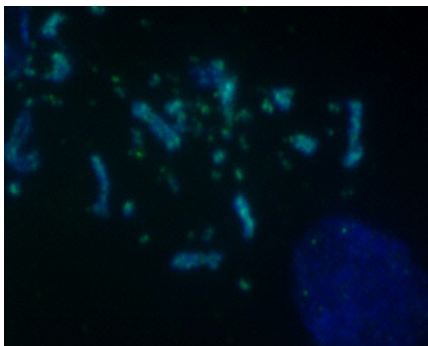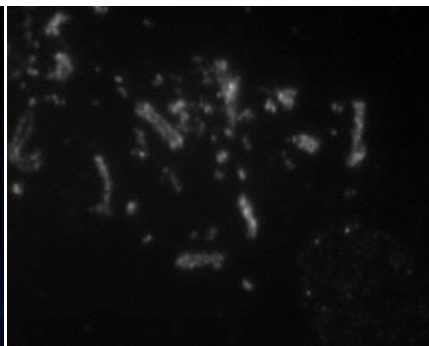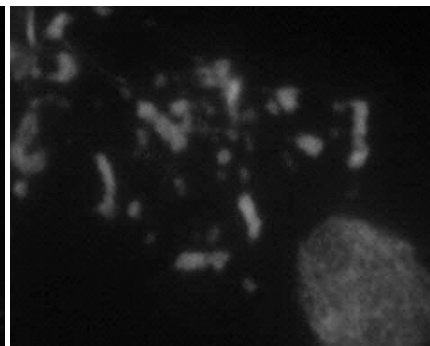

Strept488

DNA

Supplement: Additional file 1 — SMC2 and SMC2-SBP chromosome staining. (A) Top panel shows DT40 wild-type metaphase chromosomes stained with an anti-SMC2 antibody showing enrichment of the axial region of the sister chromatids. (B) Streptavidin 488 similarly binds to the axial regions of the metaphase chromosomes. [file 1471-2091-11-50-S1.PDF]

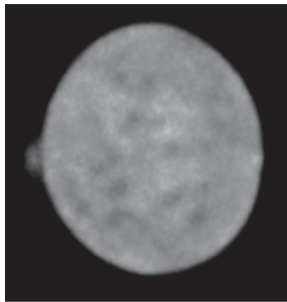

DNA

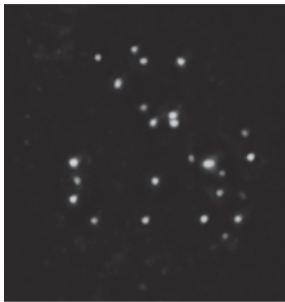

Strept488

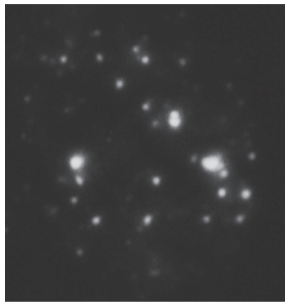

anti-Cenpa

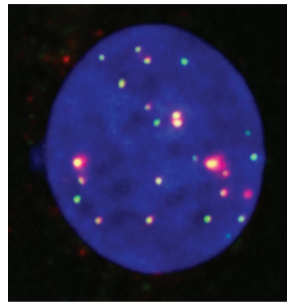

merge

Supplement: Additional file 2 — Cenpa and SBP-Cenpa interphase nuclear staining. Mouse cells transiently transfected with an SBP-Cenpa construct exhibit punctate nuclear staining detected by streptavidin-488. These signals co-localise with a rabbit antibody that is specific for the mouse Cenpa protein. [file 1471-2091-11-50-S2.PDF]

**A**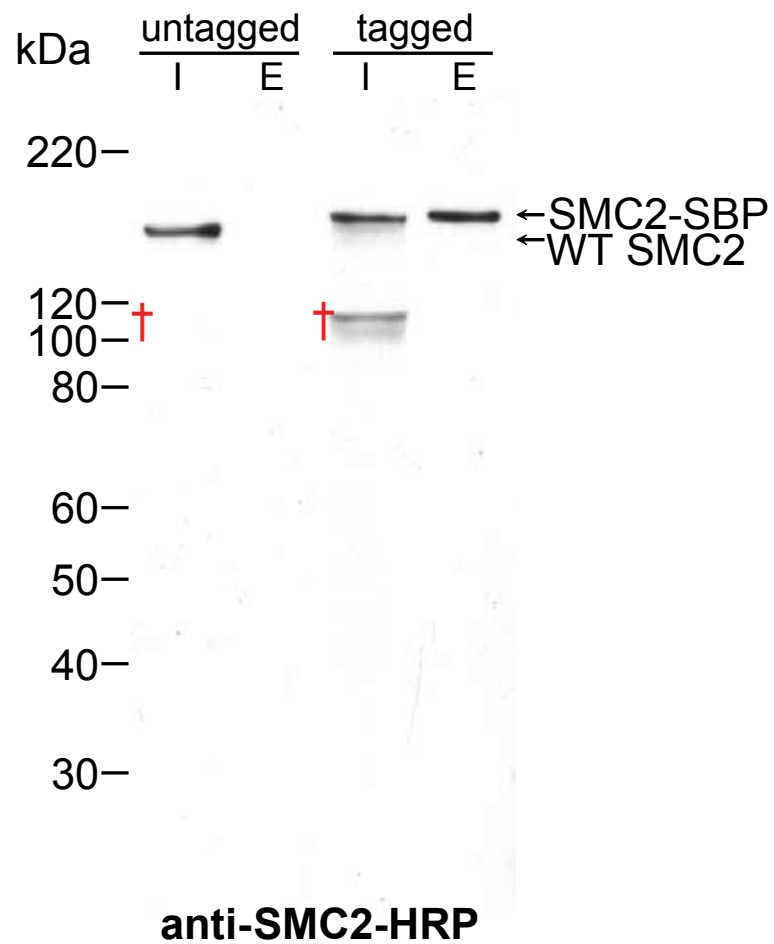**B**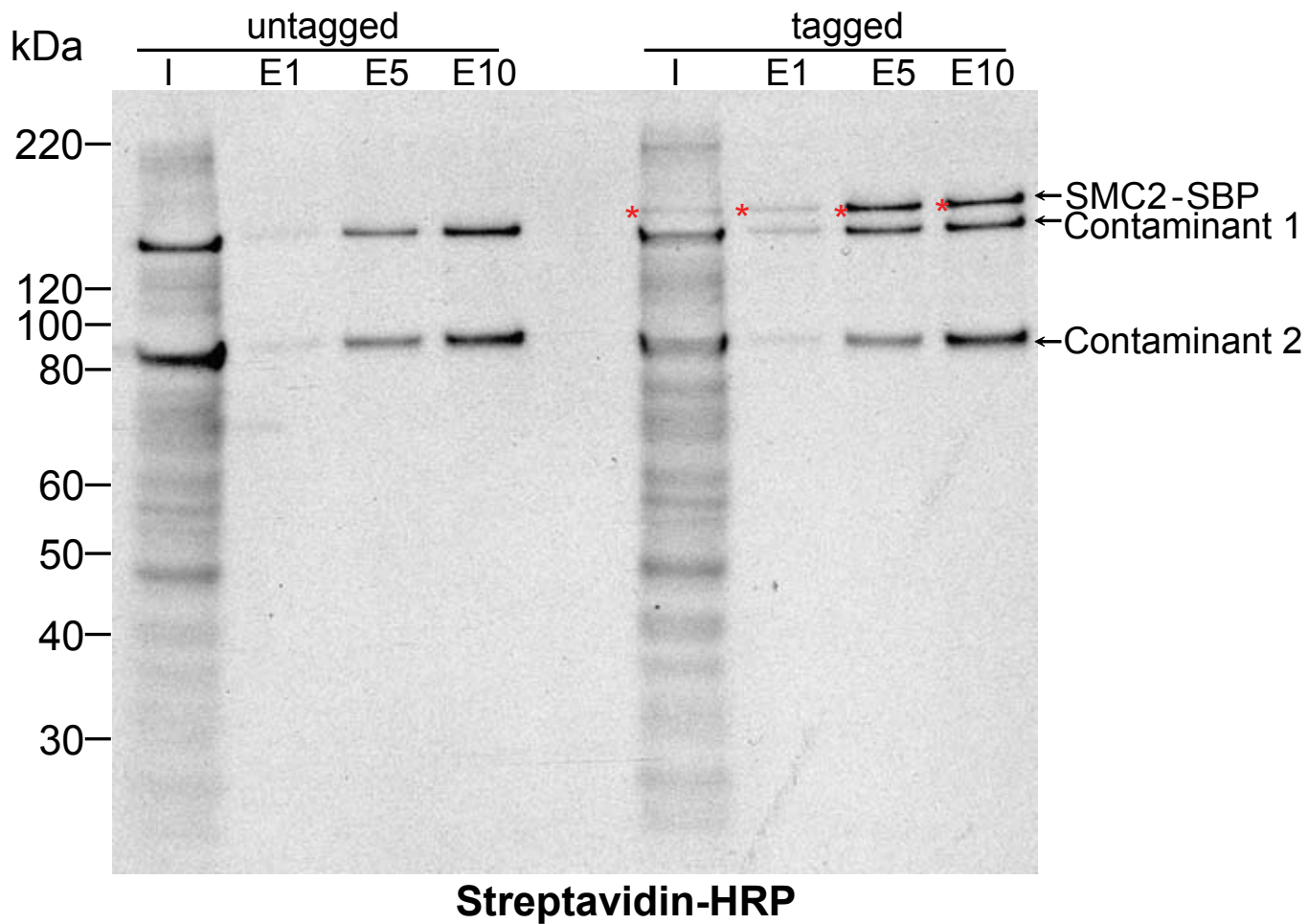

Supplement: Additional file 3 — Quantitation of SMC2-SBP purification. (A) Standardised amounts of input (I) and eluent (E) from both DT40 (untagged) and SMC2-SBP (tagged) protein extracts were immunoblotted with a rabbit anti-SMC2 antibody, followed by anti-rabbit-HRP. Similar to Figure 2B, SMC2-SBP is present in both I and E from tagged, whilst wild-type (WT) SMC2 is not present in E from untagged. Note † indicates SMC2 degradation. (B) Equivalent cell amounts (5 × 10^5^ cells) of input and eluent from both untagged and tagged (I and E1, respectively) as well as 5 and 10-fold diluted eluents (E5 and E10 respectively) were affinity blotted with streptavidin-HRP. SMC2-SBP (*) is present in all input and eluents from tagged using streptavidin HRP. Streptavidin contaminants 1 and 2 were detected in all lanes. [file 1471-2091-11-50-S3.PDF]
